# Medical innovations are driven by controversies

**DOI:** 10.1111/aogs.14373

**Published:** 2022-05-27

**Authors:** Sebastian Gidlöf, Ganesh Acharya

**Affiliations:** ^1^ Division of Obstetrics and Gynecology, Department of Clinical Science Intervention and Technology (CLINTEC) Karolinska Institutet Stockholm Sweden; ^2^ Center for Fetal Medicine Karolinska University Hospital Stockholm Sweden; ^3^ Department of Gynecology and Reproductive Medicine Karolinska University Hospital Stockholm Sweden; ^4^ Department of Clinical Medicine Women's Health and Perinatology Research Group, UiT‐The Arctic University of Norway Tromsø Norway

Acta obstetricia et gynecologica (AOGS), founded in 1921, is one of the oldest medical journals in our specialty that has been publishing innovations in obstetrics and gynecology for more than a century.^1^ A typical example is Tage Malmström's invention, the vacuum extractor, that was published in 1954, and has largely replaced forceps in Sweden and many other countries as an instrument of choice for assisted vaginal delivery.^2^ Since 1921, the science and clinical practice of obstetrics and gynecology has steadily and consistently evolved. With the rapid advances in technology, new diagnostic tools as well as medical therapies and surgical procedures have been continuously developed and tested. However, the controversies about the choice of screening, diagnostic and treatment methods continue to exist. What has been used, practiced and promoted is influenced by many factors, rather than just by the evidence available, including the availability of resources, training and skills, health economics, patient and physician preferences, local culture and traditions, as well as influence from the health policy makers, manufacturers of diagnostic and surgical equipment, and pharmaceutical companies.

We have devoted this themed issue solely to innovations and current controversies in obstetrics and gynecology. This issue includes a 10‐step approach to hysterectomy using the novel vNOTES technique and a description of a conservative approach for surgery in placenta accreta spectrum disorders. The use of mesh in vaginal prolapse surgery has lately become a controversial topic due to complications. Dr. Munch and co‐workers present patient‐reported long‐term outcomes and mesh‐related complications after treatment of apical vaginal prolapse with minimal mesh repair (Uphold). Recently, the use of vaginal laser therapy for various genito‐urinary conditions has increased. This themed issue presents a state‐of‐the‐art review on the evidence behind some of these procedures.

New innovative diagnostic techniques are continuously developed. Advances in other medical disciplines also bring new methods into obstetrics and gynecology. Accurate diagnosis and appropriate management of Injuries to the pelvic floor caused by childbirth is an emergent topic in urogynecology. As some lesions after childbirth are occult there is a clear need to investigate the role of different imaging modalities to detect these injuries and develop effective treatment methods. Lately, diagnostic methods have been refined, and in this issue of AOGS Krofta and co‐workers present a case–control study examining the use of magnetic resonance imaging in women with pelvic floor dysfunction.

There are several controversial topics in both obstetrics and gynecology, and some of them are caused by early introduction and adaptation of new technologies. For instance, the introduction of HPV vaccination programs is likely to change the spectrum of cervical dysplasia and put greater and new demands on healthcare providers. As more people are immunized, the adherence to the screening programs may decline. Bonefield et al present a pilot study investigating the use of a walk‐in‐clinic as an alternative approach to increase the uptake of the screening in non‐attenders. Eriksen and co‐workers have performed a systematic review and meta‐analysis to study the risk of recurrence in women having HPV vaccination when undergoing cone biopsies for cervical dysplasia. Furthermore, not all women have had the chance to receive HPV vaccination. Hammer et al describe active and latent HPV infection in women 50 years and older with a prior history of cervical dysplasia. HPV infection and cervical dysplasia may be worrisome to affected women. Hansen et al have performed a qualitative study to examine how women planning a pregnancy respond to active surveillance of cervical dysplasia.

Vaginal breech delivery remains a controversial topic and management differs not only between countries but also between institutions within the same country or region. Wängberg Nordborg et al present a systematic review and meta‐analysis of the differences in outcome between intended cesarean and vaginal delivery at term in women with fetus in breech presentation.

Lately, the definition of gestational diabetes has changed in many countries. Skjeldstad et al report that changing the definition and screening criteria had little impact on the prevalence of gestational diabetes in Norway. The implementation of new screening or diagnostic procedures raises new questions. Norway has only recently approved non‐invasive prenatal testing (NIPT), and Sitras et al report on controversies in implementing NIPT in the public antenatal care program.

Not only the clinical practices and research findings but also how the research data are analyzed, interpreted and presented can generate controversies. Appropriate use of statistical methods is important in this regard. Sedgwick et al have written an interesting article on null hypothesis significance testing, which we believe our readers will appreciate and enjoy reading.

Altogether, this issue presents a collection of articles describing innovations and dealing with controversial topics within the specialty of obstetrics and gynecology building on the well‐known century old tradition of AOGS. On the one hand new ideas and innovations generate controversies, and on the other hand controversies may lead to further innovations. We remain optimistic and convinced that some innovative ideas, research findings and clinical practices that are controversial today are bound to prove to be groundbreaking tomorrow providing a foundation for future evidence‐based patient‐centered healthcare.
